# Endoscopic Management of Post-Bariatric Surgery Complications: Diagnostic Work-Up and Innovative Approaches for Leak, Fistula, and Stricture Management

**DOI:** 10.3390/diagnostics16030431

**Published:** 2026-02-01

**Authors:** Jacopo Fanizza, Salvatore Lavalle, Edoardo Masiello, Francesco Vito Mandarino, Gabriele Altieri, Angelo Bruni, Francesco Azzolini, Stefano Olmi, Giovanni Carlo Cesana, Marco Anselmino, Lorenzo Fuccio, Antonio Facciorusso, Armando Dell’Anna, Mattia Brigida, Vito Annese, Silvio Danese, Sara Massironi, Gianfranco Donatelli, Giuseppe Dell’Anna

**Affiliations:** 1 Gastroenterology and Gastrointestinal Endoscopy Unit, IRCCS Policlinico San Donato, Piazza Edmondo Malan 2, 20097 San Donato Milanese, Italy; jacopo.fanizza@grupposandonato.it (J.F.); vito.annese@grupposandonato.it (V.A.); 2 Department of Medicine and Surgery, University of Enna Kore, 94100 Enna, Italy; salvatore.lavalle@unikore.it; 3 Radiology Unit, Umberto I Hospital, 94100 Enna, Italy; 4 Radiology Unit, IRCCS San Raffaele Hospital, Via Olgettina 60, 20132 Milan, Italy; 5 Gastroenterology and Gastrointestinal Endoscopy Unit, IRCCS San Raffaele Hospital, Via Olgettina 60, 20132 Milan, Italy; mandarino.francesco@hsr.it (F.V.M.); altieri.gabriele@hsr.it (G.A.); azzolini.francesco@hsr.it (F.A.); danese.silvio@hsr.it (S.D.); sara.massironi@grupposandonato.it (S.M.); 6 Faculty of Medicine and Surgery, Vita-Salute San Raffaele University, Via Olgettina 56, 20132 Milan, Italy; stefano.olmi@grupposandonato.it (S.O.); giovanni.cesana@grupposandonato.it (G.C.C.); 7 Gastroenterology Unit, Department of Medical and Surgical Sciences, IRCCS Azienda Ospedaliero-Universitaria di Bologna, University of Bologna, 40100 Bologna, Italy; angelo.bruni4@unibo.it (A.B.); lorenzo.fuccio3@unibo.it (L.F.); 8 Department of General and Mini-Invasive Surgery, Policlinico San Marco, 24040 Zingonia, Italy; 9 Bariatric Surgery Unit, IRCCS San Raffaele Hospital, Via Olgettina 60, 20132 Milan, Italy; anselmino.marco@hsr.it; 10 Gastroenterology Unit, Faculty of Medicine and Surgery, University of Salento, Piazza Tancredi 7, 73100 Lecce, Italyarmando.dellanna@alice.it (A.D.); mattiabrigida@hotmail.it (M.B.); 11 Digestive Endoscopy Unit, “Vito Fazzi” Hospital, Piazza Filippo Muratore 5, 73100 Lecce, Italy; 12 Department of Clinical Medicine and Surgery, University of Naples “Federico II”, 80138 Naples, Italy; gianfranco.donatelli@unina.it; 13 Unité d’Endoscopie Interventionnelle, Hôpital Privé des Peupliers, Ramsay Générale de Santé, 75013 Paris, France

**Keywords:** bariatric surgery, sleeve gastrectomy, leak, stricture, fistula, endoscopic vacuum therapy, SEMS, EVT, endoscopic internal drainage

## Abstract

Bariatric surgery is an effective treatment for morbid obesity but is frequently complicated by anastomotic leaks, fistulas, and strictures, which can significantly impair patient outcomes. Optimal management of these complications relies on a timely and accurate diagnostic assessment; however, effective treatment strategies are central to improving clinical recovery. This review primarily focuses on the endoscopic management of post-bariatric surgery complications, while providing a concise overview of the diagnostic imaging modalities that guide therapeutic decision-making. Contrast-enhanced imaging techniques, including computed tomography (CT) and fluoroscopy, as well as endoscopic ultrasound (EUS), are briefly discussed in relation to their role in identifying complications, defining their extent, and selecting the most appropriate endoscopic intervention. The core of this review is dedicated to current endoscopic treatment approaches, including endoscopic internal drainage with double pigtail plastic stents, self-expanding metal stents (SEMSs), endoscopic vacuum therapy (EVT), and EUS-guided drainage of fluid collections. Particular emphasis is placed on indications, technical considerations, and outcomes of these therapies. Finally, this review highlights emerging endoscopic technologies that may further optimize the management of post-bariatric surgery complications and improve patient outcomes, underscoring the evolving role of minimally invasive endoscopic treatment within a multidisciplinary framework.

## 1. Introduction

Obesity has reached alarming proportions worldwide, posing a substantial challenge to global healthcare systems [1]. Its health consequences are severe and wide-ranging, including an increased risk of cardiovascular diseases, type 2 diabetes mellitus, musculoskeletal disorders, and certain types of cancer [2]. The persistent rise in the prevalence of obesity necessitates the implementation of effective interventions to mitigate their impact on public health. In this context, several current treatment options for obesity include dietary interventions, pharmacological therapy, endoscopic procedures, and bariatric surgery [3]. In this context, bariatric surgery has emerged as a promising therapeutic strategy for individuals with severe obesity, offering significant weight loss and improvements in obesity-related comorbidities [4]. Among the most commonly performed procedures are Roux-en-Y gastric bypass (RYGB) and sleeve gastrectomy (SG), with SG surpassing RYGB in 2013 to become the most frequently performed bariatric surgery worldwide, owing to its effectiveness and relatively lower invasiveness [4]. Although postoperative mortality from bariatric surgery is low, less than 1% [5], patients often require urgent medical care following the procedure. More than half of patients will seek urgent care at least once within the first two years for bariatric-related issues, with up to 29% needing such care within the first 90 days post-surgery [6]. Approximately 10% of these visits result in hospital readmission due to surgical complications [6]. The 30-day complication rate is 5.8% for laparoscopic SG and 8.0% for RYGB [7]. Complications following bariatric surgery can be classified into early (occurring immediately after surgery) and late (developing after 30 days) complications [8]. Early complications include infection, hemorrhage, anastomotic leakage, pulmonary embolism, and perforation. Late complications, which arise beyond the immediate postoperative period, encompass nutritional deficiencies, gallstone formation, gastrointestinal ulcers, internal hernias, and stenosis [8]. Given the wide range of potential complications, close monitoring in the immediate postoperative period is essential. Moreover, upon the emergence of clinical suspicion, a prompt diagnostic workup should be initiated in order to achieve the earliest possible diagnosis. Patient assessment begins with a thorough surgical history, as identifying the type of procedure and the timing of its execution is crucial for determining the potential complications and the appropriate diagnostic investigations [9]. Physical examination should prioritize identifying vital sign abnormalities such as fever, tachycardia, tachypnea, and hypoxia. For example, tachycardia or tachypnea within the first postoperative month may indicate an anastomotic leak or pulmonary embolism [10]. Laboratory workup should include a complete blood count with differential, comprehensive metabolic panel, lactate, C-reactive protein, coagulation profile, and blood type and crossmatch if gastrointestinal bleeding is suspected. Blood and urine cultures should be considered in septic or hemodynamically unstable patients [10]. Clinical signs are frequently absent or nonspecific, and symptoms may be intermittent and difficult to detect during evaluation. Abdominal pain, a primary complaint following bariatric surgery, can indicate a spectrum of conditions from benign to potentially life-threatening [7,11]. Although over half of patients with anastomotic dehiscence report abdominal pain, physical examination may not reveal tenderness, likely due to factors such as the substantial subcutaneous tissue in obese patients, subphrenic localization of intraperitoneal infection, and altered inflammatory responses [7,11]. Additionally, abdominal pain, particularly in the epigastric region, is common in patients who develop anastomotic stenosis, such as after RYGB; in these cases, assessing accompanying symptoms like dysphagia or vomiting is crucial [12]. Due to the altered postoperative anatomy in bariatric surgery patients, selecting the appropriate imaging modality is crucial for accurate diagnosis. In the context of suspected postoperative complications, oral contrast administration during CT imaging is essential. Oral contrast significantly enhances the visualization of key anatomical structures, such as the gastric pouch, gastrojejunostomy, Roux limb, jejunojejunostomy, and biliopancreatic limb, thus aiding in the diagnosis of internal hernias and small bowel obstructions [13]. Furthermore, the detection of anastomotic leaks is notably improved when both oral and IV contrast are used. Upper gastrointestinal (GI) fluoroscopy and contrast-enhanced CT are the primary imaging tools for detecting anastomotic leaks and are often complementary. Studies show that the combination of both techniques (detection rate approximately of 70% vs. 56% of CT IV alone) improves diagnostic accuracy compared to either alone [14]. While combining upper GI series with CT may optimize diagnostic performance, it is not always feasible in all emergency settings. Thus, a CT with both oral and IV contrast is a practical and effective initial approach, though clinicians should remain aware of the risk of false negatives in non-specialized centers. In high-volume bariatric centers, CT has shown up to 95% sensitivity and 100% specificity for detecting anastomotic leaks, suggesting its use as the optimal first-line diagnostic tool [15]. Endoscopic techniques are frequently utilized to address complications following bariatric surgery, as they offer a less risky alternative to reoperation. Endoscopy plays a crucial role in both diagnosing and managing these postoperative issues [16]. However, effective management typically involves a multidisciplinary team, which may include gastroenterologists, bariatric surgeons, surgical endoscopists, interventional radiologists, and registered dietitians. This review focuses on the diagnostic evaluation of complications following bariatric surgery, with particular attention to leaks, fistulas and strictures. Imaging modalities, notably contrast-enhanced CT and endoscopic ultrasound (EUS), play a pivotal role in differentiating key complications such as leaks, fluid collections, obstructions, and strictures, as well as in assessing the extent of anatomical and functional damage. These diagnostic tools not only facilitate timely and accurate identification but also aid in guiding therapeutic decisions. Building upon this diagnostic framework, this review discusses principal endoscopic interventions, including internal drainage with double pigtail plastic stents, self-expanding metal stents (SEMSs), endoscopic vacuum therapy (EVT), and EUS-guided drainage of perivisceral collections, emphasizing their role within the diagnostic pathway. Importantly, standardized guidelines for the diagnostic and therapeutic management of post-bariatric complications are currently lacking, with clinical strategies largely dependent on institutional expertise and multidisciplinary collaboration [17]. Finally, this review highlights emerging technologies poised to enhance diagnostic precision and foster more personalized, minimally invasive management of these complex post-surgical conditions.

## 2. Materials and Methods

In our review, we performed a thorough literature search using PubMed, Scopus, Web of Science, and Medline, focusing on English-language articles published up to the end of June 2025. Our search strategy employed comprehensive search strings that included terms such as “bariatric surgery,” “sleeve gastrectomy,” “leak,” “fistula,” “endoscopic vacuum therapy,” “Roux-en-Y gastric bypass,” “stricture,” “self-expandable metal stents,” “endoscopic internal drainage,” and “collections.” We also conducted manual screening of the reference lists from the included studies and relevant reviews to identify additional eligible publications.

## 3. Radiological Assessment of Post-Bariatric Surgery Complications

### 3.1. Indications for Imaging in Post-Bariatric Patients

Postoperative bariatric patients require prompt imaging evaluation whenever complications are suspected, as early diagnosis is critical to reducing morbidity. Indications for imaging include any clinical signs of an anastomotic leak (such as unexplained tachycardia, fever, severe abdominal or chest pain, and oliguria) within the first days after surgery, signs of sepsis or peritonitis, or persistent postoperative ileus beyond the expected recovery period. In the early postoperative phase (generally within 10–14 days), a leak is one of the most feared complications and typically presents with acute abdominal pain, fever, tachycardia, and often hypotension [18]. Any such presentation warrants immediate imaging to confirm a leak or identify intra-abdominal collections. Similarly, unexplained abdominal pain in later postoperative periods raises concern for internal hernia in RYGB patients or strictures causing obstruction in sleeve gastrectomy patients. For instance, months to years after RYGB, intermittent colicky pain, postprandial vomiting, or signs of small-bowel obstruction are red flags for an internal hernia through a mesenteric defect [19].

### 3.2. Imaging Modalities: Protocols and Comparative Utility

Multiple imaging modalities are employed in evaluating post-bariatric surgery patients, each with distinct protocols and utilities. The principal modalities are fluoroscopic upper gastrointestinal (UGI) contrast studies and abdominal CT scans, which together form the mainstay of postoperative imaging [20]. Traditionally, fluoroscopy (UGI series or swallow study) has been the accepted first step for evaluating the surgical anatomy, checking for obstructions or strictures, and detecting leaks by oral contrast extravasation. With the rise of cross-sectional imaging, however, contrast-enhanced CT has assumed a central role due to its superior specificity in characterizing major complications [21]. In practice, these modalities are often complementary: an UGI study can directly visualize luminal continuity and leak sites, while CT scan survey the extraluminal spaces for subtle leaks, abscesses, or internal hernias. Ultrasound (US) is likewise of limited diagnostic yield in this setting due to body habitus and deep location of surgical sites; its use is mostly confined to screening for unrelated issues. US may identify superficial fluid collections or incisional hernias but cannot reliably evaluate intraperitoneal complications after bariatric surgery [22].

#### 3.2.1. Plain Radiography

Abdominal X-rays plays a limited role—it is highly non-specific and thus seldom performed in the routine postoperative evaluation. That said, simple radiographs can occasionally provide clues: for example, free subdiaphragmatic air on an upright chest/abdominal X-ray may signal a viscus perforation or leak (though some free air is expected immediately after laparoscopic surgery), and abnormal bowel gas patterns or multiple air-fluid levels may suggest an obstruction [23]. In patients with an adjustable gastric band, a plain radiograph can confirm band position and orientation; an acute band slippage may be evident as an abnormal band angle or prolapse of the stomach above the band on X-ray [24].

#### 3.2.2. Fluoroscopic Contrast Studies

**Figure 1:**
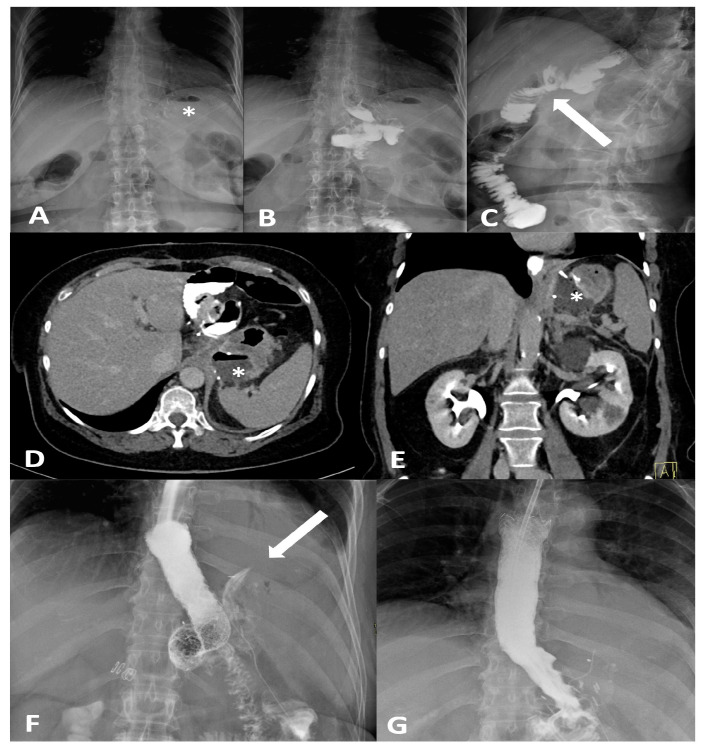
Frontal (**A**,**B**) and oblique (**C**) X-ray for postoperative bariatric surgery assessment. Surgical clips (asterisk) are visible in the epigastric region and a surgical drain is present in the left hypochondrium. The examination was performed following oral administration of water-soluble contrast agent. The esophagus is patent, with normal caliber and course. The gastrojejunostomy (arrow) shows regular opacification. No extraluminal contrast leakage is observed. Opacified small-bowel loops are seen, without evidence of dilatation. Four days later, following the onset of fever and abdominal pain, the patient was re-evaluated with CT. CT equilibrium-phase axial (**D**) and coronal (**E**) scans showing postsurgical changes from gastric bypass with creation of an L–L gastrojejunostomy. Between the medial contour of the gastric remnant and the gastric pouch—inseparable from the left diaphragmatic crus, which appears thickened and slightly heterogeneous—a fluid-containing collection (asterisk) is visible. The adjacent adipose tissue demonstrates a heterogeneous appearance, with several small air bubbles present within it. After the placement of an esophageal stent for protection, the follow-up X-ray with oral contrast (**F**) shows extraluminal leakage of contrast (arrow) originating from the mid portion of the stent, opacifying a retrogastric collection, as also visible on the CT scans. Ten days later, after placement of a nasojejunal tube, the X-ray with oral contrast (**G**) no longer shows the previously reported extraluminal leakage. The copyright of this figure belongs to the authors.

Fluoroscopic UGI contrast studies are a cornerstone for evaluating the gastric pouch, anastomoses, and sleeve lumen. The protocol involves obtaining an initial abdominal scout radiograph followed by oral ingestion of ~50–100 mL of a dilute water-soluble contrast agent (e.g., diatrizoate meglumine and diatrizoate sodium) [25]. Real-time fluoroscopy is used to observe the passage of contrast through the esophagus and the new gastric anatomy into the small bowel. For RYGB patients, the contrast column is typically followed all the way through the Roux limb to the jejunojejunal anastomosis and into distal small bowel to ensure no distal obstruction [26]. If no leak is seen with water-soluble media, many protocols then administer high-density barium to obtain better mucosal coating and detail of the lumen. Important safety caveats are observed: barium is avoided initially if a leak is suspected, since extravasated barium can cause severe chemical peritonitis [27]. Instead, water-soluble contrast is used for leak testing, and only if the study is negative for leak is barium employed to evaluate strictures or anatomy in fine detail [28]. Fluoroscopy’s advantages are its ability to directly visualize anastomotic strictures (as a fixed narrowing with proximal dilation and delayed transit) and to catch leaks dynamically. A classic fluoroscopic finding of a leak is the appearance of contrast extravasation as a tracking collection or free spillage outside the GI tract—for example, a sleeve gastrectomy leak often shows contrast pooling just lateral to the staple line near the gastroesophageal junction [29]. In RYGB, small leaks at the gastrojejunostomy may appear as tiny sinus tracts or outpouchings of contrast adjacent to the anastomosis on certain views [30] (Figure 1). The sensitivity of fluoroscopy for leaks is good for gross leaks, but small or intermittent leaks can be missed, especially if they drain externally or if patient positioning is suboptimal. Thus, a negative UGI does not fully exclude a leak in a symptomatic patient—continued high suspicion warrants further imaging by CT [31]. For detecting strictures, the UGI series is very useful: a stricture after sleeve gastrectomy may be seen as a hold-up of contrast with a long narrow segment at the incisura or distal sleeve, whereas an RYGB gastrojejunostomy stricture appears as a smooth, tapered narrowing at the stoma with delayed emptying of the gastric pouch [32]. Fluoroscopy can also demonstrate functional issues such as reflux or outlet obstruction by showing refluxed contrast or failure of contrast to pass despite peristalsis [33].

#### 3.2.3. Computed Tomography

**Figure 2:**
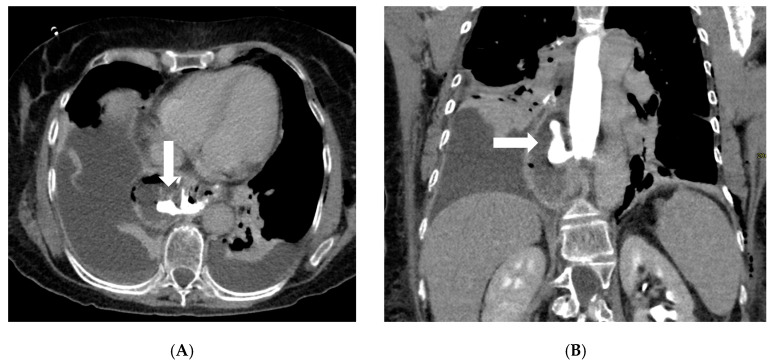
Axial (**A**) and coronal (**B**) post-contrast CT scans in a patient on postoperative day 3 following gastric bypass with esophagojejunostomy positioned in the mediastinum (history of sleeve gastrectomy). An esophagojejunal feeding tube is in place. To the right of the esophagojejunal anastomosis, a hypodense collection with internal air components is visible. After oral administration of water-soluble contrast, a posterior dehiscence of the anastomosis is demonstrated, with extraluminal contrast leakage accumulating within the previously described collection (arrows). The copyright of this figure belongs to the authors.

Computed tomography (CT) with oral and IV contrast is the workhorse for diagnosing post-bariatric complications, especially when the clinical picture is severe or the fluoroscopic exam is inconclusive [34]. Modern multidetector CT allows excellent visualization of both intraluminal detail (via oral contrast) and extraluminal pathology (via IV contrast enhancement and high-resolution imaging) [35]. Protocols typically include administration of water-soluble oral contrast before scanning—either given enterally a short time prior or even in the CT suite just before the scan to opacify the gastric pouch/sleeve and bowel—combined with IV contrast in the portal venous phase. A non-contrast (baseline) CT scan is essential in bariatric surgery imaging protocols when the identification of surgical materials is required (Table 1). This phase allows for the optimal visualization of high-density materials such as surgical staples, clips, gastric bands, and synthetic meshes, which may otherwise be obscured or confused with enhanced vascular or pathological structures in post-contrast imaging (Figure 2).

**Figure 3:**
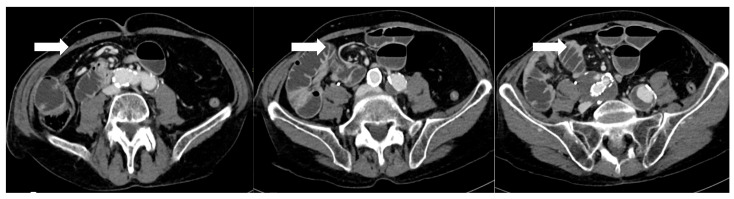
After 5 days from sleeve gastrectomy, axial post-contrast CT scans showing an angulated course of the duodenojejunal junction to the right of the midline, with evidence of a mesenteric “whirlpool sign,” suggestive of intestinal malrotation (arrows). The copyright of this figure belongs to the authors.

CT’s strengths lie in its high sensitivity for extraluminal gas and fluid, its ability to detect subtle signs like focal mesenteric fat stranding or free air under the diaphragm (which on CT can be discerned even in small volumes), and its capacity to evaluate the entire abdomen for alternative diagnoses. CT can also identify complications like small bowel obstruction from adhesions or hernias, and vascular problems (e.g., splenic infarcts from short gastric artery injury or portal vein thrombosis), which occasionally occur after bariatric surgery [20]. If active bleeding is suspected (for example, a late marginal ulcer hemorrhage or bleeding at a staple line), a CT angiography protocol can be employed to look for contrast extravasation in arterial phase; this can guide interventional radiology if an embolization is needed [36]. One significant strength of CT is in diagnosing internal hernias and small-bowel obstructions, particularly in RYGB patients. An internal hernia occurs when bowel loops protrude through a mesenteric defect created by the surgery (such as the transverse mesocolon defect in a retrocolic Roux limb, the mesenteric gap at the jejunojejunostomy, or Petersen’s space between the Roux limb and transverse colon mesentery) [37]. Classic CT signs of internal hernia after RYGB include a swirled appearance of the mesenteric root fat and vessels (the “whirl” or swirl sign), which reflects the twisting of mesentery as bowel herniates [38] (Figure 3). Other CT hallmarks are clustered, ectopic small-bowel loops (often congregated in an abnormal location such as the left upper quadrant or behind the mesenteric root) with evidence of obstruction (dilated proximal loops and decompressed distal bowel) [39]. Additional reported signs include engorged and stretched mesenteric vessels converging toward the hernia orifice, beaking and crowding of the superior mesenteric vein, the “hurricane eye” sign (a tubular cluster of mesenteric fat surrounded by bowel loops on cross-section), and displacement of the normal jejunojejunal anastomosis position [40]. However, a study by Lockhart et al. and subsequent analyses indicated that mesenteric swirl carries the highest sensitivity and specificity for internal hernia and should be considered pathognomonic in the appropriate clinical context [41]. CT is equally valuable in characterizing fistulous connections and differentiating them from free leaks. For example, a known late complication of RYGB is the formation of a gastro-gastric fistula (GGF), wherein a communication develops between the gastric pouch and the excluded stomach due to staple-line dehiscence. On a CT with oral contrast, a GGF is diagnosed when contrast that was swallowed (entering the pouch) is seen opacifying the excluded stomach, while notably not filling the downstream biliopancreatic limb or duodenum [42].

#### 3.2.4. Magnetic Resonance Imaging

Magnetic Resonance Imaging (MRI) is not routinely used for acute post-bariatric complications, but it can be invaluable in scenarios where radiation or contrast exposure should be minimized—notably in pregnant patients or those with severe IV contrast allergy or renal failure. For pregnant patients with a history of RYGB who present with abdominal pain, MRI offers a safe alternative to CT for diagnosing internal hernia. Studies have shown that MRI can approach the diagnostic performance of CT in this context. For example, an MRI (using rapid T2-weighted sequences like HASTE) can demonstrate the mesenteric swirl sign and dilated loops characteristic of an internal hernia, like CT [43]. More recently, a 2022 single-center study using a fast T2-sequence MRI in pregnant RYGB patients found excellent sensitivity and negative predictive value for identifying internal herniation, supporting MRI as a viable emergency imaging modality in this population [44]. Thus, MRI has emerged as the preferred imaging for suspected internal hernia in pregnancy, avoiding X-rays altogether while still providing diagnostic confidence [44]. MRI can visualize signs like engorged, mispositioned bowel loops and abnormal mesenteric positioning without contrast, and adding an MR angiography sequence can even evaluate bowel perfusion if ischemia is a concern [45]. Outside of pregnancy, MRI is occasionally used to evaluate postoperative hepatic or biliary issues (for instance, an MRCP to assess the biliary tree in a RYGB patient with suspected CBD stones, since ERCP is difficult) [46].

## 4. Epidemiology, Risk Factors and Clinical Features

### 4.1. Epidemiology, Risk Factors and Clinical Features of Leaks and Fistulas After SG and RYGB

Anastomotic or staple-line leaks are a rare but potentially life-threatening complication following bariatric surgery, particularly after SG and RYGB. Although the incidence of postoperative gastrointestinal leaks has declined over time, it remains a relatively rare complication associated with considerable morbidity and mortality [47]. The overall incidence of anastomotic leaks after bariatric surgery is reported to range between 1% and 6%. The incidence of leaks following RYGB has been reported to be as high as 8.3%; however, more recent data indicate a rate closer to 1.1% [48]. In comparison, the incidence of leaks or fistulas after SG is estimated to occur in approximately 2% to 5% of cases [49,50]. The overall mortality rate related to leaks in RYGB is relatively low, at 0.6%. However, the mortality specifically among patients who experience a leak is significantly higher, ranging from 14.7% to 17% [51]. Similar patterns are observed in SG, where the overall leak-related mortality of 0.14% and a leak-associated mortality rate of 9% [52]. These leaks most commonly occur at the proximal staple line just below the angle of His in SG, and at the gastrojejunal (GJ) anastomosis in RYGB [53]. In SG, to avoid stapling too close to the esophagus, surgeons may intentionally leave a small fundal pouch which may predispose to ischemia due to division of the short gastric arteries. Additional contributing factors include inappropriate staple height selection owing to the thin gastric wall in the fundus, downstream stenosis at the incisura angularis, and elevated intraluminal pressure within a noncompliant gastric sleeve [17,54]. As a result, most leaks are not present at the conclusion of the procedure but tend to manifest in the ensuing weeks, frequently in association with distal obstruction. Gastrointestinal leaks occurring within the first 48 h after surgery are typically linked to mechanical factors, such as technical issues during the procedure. In contrast, leaks that arise more than 5–6 days postoperatively are more often attributed to ischemic mechanisms [55,56]. According to the Rosenthal classification, leaks are categorized based on the time of onset into acute (≤1 week), early (1–6 weeks), late (6–12 weeks), and chronic (>12 weeks), a framework that aids in guiding diagnostic and therapeutic strategies [57]. An alternative classification of leaks following SG is based on their location and severity. Type I, or subclinical leaks, are confined and do not present with sepsis or extension into the abdominal or pleural cavities. In contrast, type II leaks, referred to as clinical, are associated with septic symptoms and extensive spread within the abdominal or pleural spaces [58,59]. Several studies have investigated the predictive factors for anastomotic leaks following RYGB. An analysis based on the large MBSAQIP database identified numerous independent preoperative risk factors [60]. Among these, a high body mass index (BMI), advanced age, an ASA score ≥ 3, a history of pulmonary embolism, and a partially dependent functional status were significantly associated with an increased risk of leak. Conversely, higher preoperative albumin levels, indicative of better nutritional status, and longer operative duration, likely reflecting greater technical precision, were identified as protective factors against the occurrence of postoperative complications [60]. Another study confirmed and expanded on these findings, highlighting additional risk factors including comorbidities such as obstructive sleep apnea, hypertension, diabetes mellitus, and hypoalbuminemia [61] (Figure 3). Interestingly, certain intraoperative practices, such as the use of provocative tests to assess anastomotic integrity and the placement of surgical drains, were associated with an increased incidence of fistulas. In contrast, the implementation of a postoperative swallow study showed no significant impact on the early detection of leaks [61]. Clinically, early recognition of leaks remains essential. Symptoms such as persistent tachycardia (heart rate > 90 bpm), fever, increased intravenous fluid requirements, and a rapid rise in C-reactive protein (CRP) levels from as early as postoperative day two are considered reliable warning signs. In some cases, these clinical indicators may precede radiological evidence, underscoring the importance of careful clinical monitoring in the days following surgery [60]. Regarding SG, male sex, elevated waist circumference, hypoproteinemia, and type 2 diabetes have been recognized as risk factors for postoperative leaks (Figure 1). Preventing acute leaks requires optimization of the surgical technique, specifically through meticulous dissection of the angle of His and conserving a smaller segment of the gastric fundus [62]. A recent meta-analysis identified several significant risk factors for the development of postoperative leaks following SG and RYGB [63]. Cigarette smoking was associated with an increased risk of leaks in SG patients, with an odds ratio (OR) of 1.71 [1.44, 2.05], whereas in RYGB patients the risk increase was less pronounced and not statistically significant (OR 1.09 [0.82, 1.42]). When combining both procedures, the overall risk remained elevated (OR 1.31 [1.06, 1.61]) [63]. DM did not significantly increase leak risk after SG (OR 1.11 [0.97, 1.28]), but a significant effect was observed in RYGB patients (OR 1.33 [1.02, 1.73]); combining data from both procedures yielded an OR of 1.19 [1.11, 1.28]. Hypertension, initially not associated with increased risk, was subsequently identified as a moderate risk factor with a risk ratio of 1.17 [1.10, 1.24]. The most prominent risk factor was chronic kidney disease (CKD), which conferred a significantly increased risk of leak, with an OR of 2.41 [1.42, 3.99]. Finally, chronic corticosteroid therapy was also associated with an increased risk of postoperative leaks (OR 1.57 [1.22, 2.02]) [63] (Figure 1).

### 4.2. Epidemiology, Risk Factors and Clinical Features of Stenosis After SG and RYGB

**Figure 4:**
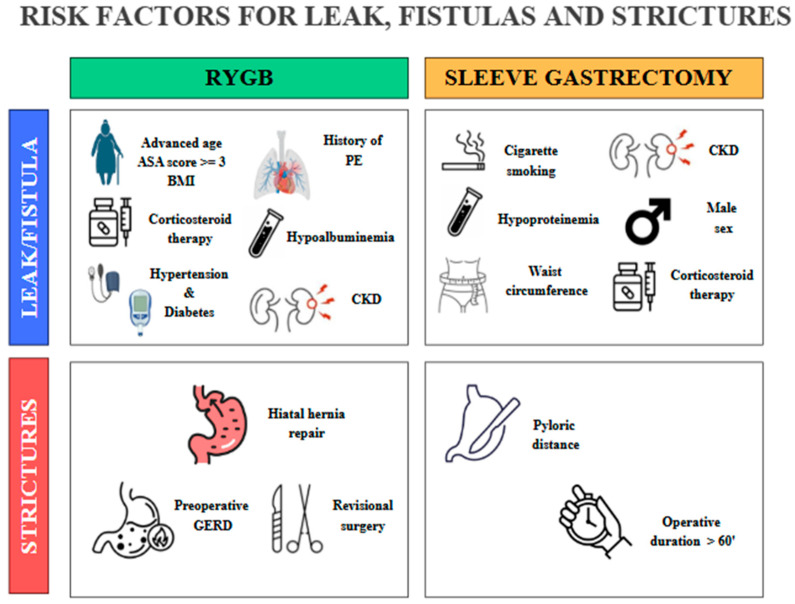
Some preoperative and procedure-related risk factors involved in the development of leaks, fistulas, and strictures after RYGB and SG. PE, pulmonary embolism; CKD, chronic kidney disease; GERD, gastroesophageal reflux disease. The copyright of this figure belongs to the authors.

Anastomotic stenosis following RYGB occurs in approximately 3–27% of patients, most frequently at the gastrojejunal (GJ) anastomosis. In contrast, strictures at the jejunojejunal anastomosis are rare, with an incidence of around 0.5% [64]. Gastric sleeve stenosis has been reported in 0.5–3.5% of cases [65] and typically develops due to the progressive twisting of the staple line and scarring, which causes the sleeve to become kinked. It can also result from imbrication of the staple line or excessive retraction of the greater curvature during stapling. The most common locations for stenosis are the incisura angularis and the gastroesophageal junction [66]. This complication is often associated with proximal sleeve leaks, likely resulting from increased intraluminal pressure on the staple line and subsequent upstream dilation [65]. In SG, two technical factors have been independently associated with a higher risk of stricture formation: operative duration exceeding 60 min (OR 1.32; 95% CI: 1.17–1.48; *p* < 0.001), and a shorter distance from the pylorus to the start of gastric transection (pyloric distance, PD), with each additional centimeter of PD associated with a 13.6% reduction in stricture risk (OR 0.86; 95% CI: 0.78–0.95; *p* = 0.003) [67] (Figure 4). Conversely, other intraoperative variables such as bougie size, staple line reinforcement, and oversewing have not demonstrated significant associations with stenosis (*p* > 0.05) [67]. For RYGB procedures, several independent risk factors for early GJ stricture have been identified [68]. These include concurrent hiatal hernia repair (OR 1.8; 95% CI: 1.5–2.2; *p* < 0.001), revisional surgery (OR 1.4; 95% CI: 1.1–1.6; *p* = 0.001), and preoperative gastroesophageal reflux disease (GERD) (OR 1.4; 95% CI: 1.2–1.5; *p* < 0.001) [68] (Figure 1). Additionally, the placement of surgical drains (OR 1.3; 95% CI: 1.1–1.4; *p* = 0.001) and the routine use of postoperative contrast swallow studies (OR 1.3; 95% CI: 1.1–1.5; *p* = 0.001) have been associated with a significantly increased risk of developing early GJ strictures [68].

A 2021 meta-analysis conducted by Yi-Shyue Chen et al. evaluated the effectiveness of fibrin sealants in preventing complications after bariatric surgery, particularly leaks and strictures. Fibrin sealants, composed of fibrinogen and thrombin, replicate the final step of the coagulation cascade, providing early hemostasis [69]. They act not only as hemostatic agents but also as a provisional, remodelable matrix that supports the healing and fibrotic processes. In practice, they function like an adhesive network that holds cells and clot components together, helping to prevent the leakage of gas or fluids [70]. Among the most used products, Tisseel (Baxter Inc., USA) was approved by the FDA in 1998, while other commercially available formulations include Evicel (Ethicon Inc., USA), Crosseal (Omrix Biopharmaceuticals Ltd., Israel), and Hemaseel (Heamacure Corp., Canada) [71]. Therefore, perioperative use of fibrin sealants has been proposed as a preventive strategy to reduce post-surgical complications. The combined data showed a trend toward a lower incidence of leaks in the fibrin sealant group compared to the control group, as well as a slightly higher incidence of gastric strictures. However, these differences were not statistically significant. Overall, the results suggest that fibrin sealants may provide valuable perioperative support, although current evidence does not demonstrate a clear or consistent benefit in preventing complications [69]. The clinical presentation of SG strictures closely resembles that of a gastric outlet obstruction. Patients commonly report food intolerance, nausea, vomiting, dysphagia, and postprandial abdominal pain. Additionally, due to the loss of normal gastric accommodation following SG, gastroesophageal reflux and regurgitation are frequently observed [72]. The symptoms associated with strictures after RYGB are similar to those seen in SG strictures, with dysphagia, intolerance to solid foods, nausea, and vomiting being the most frequently reported. Other symptoms—such as abdominal pain, odynophagia, or gastroesophageal reflux—are less common but should still be considered during the comprehensive clinical assessment, as they may indicate the presence of a marginal ulcer or other underlying abnormalities [73].

## 5. Management of Complications

### 5.1. Anastomotic and Staple Line Leaks Management

#### 5.1.1. Endoscopic Closure Techniques: Over-the-Scope Clip and Endoscopic Suturing

##### Over-The-Scope Clip

**Figure 5:**
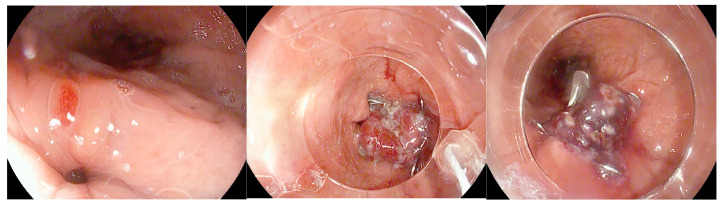
Gastric fistula following sleeve gastrectomy that was treated by placement of an Over-The-Scope Clip. The copyright of this figure belongs to the authors.

Over the years, the therapeutic approach to leaks has traditionally relied on percutaneous drainage, performed through interventional radiology or surgery, followed by endoscopic attempts at closure. Among the most frequently employed techniques are endoscopic suturing and the Over-The-Scope Clip (OTSC) [17]. However, clinical experience has shown that recurrence of the leak is common when these primary closure methods are used in isolation. The OTSC system (Ovesco Endoscopy, Tübingen, Germany) is composed of a large Nitinol clip that is preloaded onto the distal tip of the endoscope (Figure 5) [74].

Endoscopic closure tends to be more effective when the surrounding tissue maintains sufficient integrity without significant ischemia or inflammation, and when the endoscopist can obtain a perpendicular view of the defect to ensure precise repair. Equally important is the management of elevated intraluminal pressures, which may otherwise persist due to downstream gastric or anastomotic stricture [75]. However, evidence on the efficacy of OTSC in the management of postbariatric leaks and fistulas is sparse. Keren et al. published a retrospective study representing the largest case series to date of patients with leaks following SG treated with the OTSC system [76]. The study included 26 patients. 16/26 patients (61.5%) underwent primary sleeve gastrectomy, while 10/26 (38.5%) had undergone bariatric revision procedures. Endoscopic management typically required multiple sessions, ranging from two to seven per patient, with the majority being successfully treated using a single clip; a few cases necessitated the placement of a second clip. Some patients also received adjunctive therapies, such as stents, biological glues, or biliary prostheses, to facilitate closure. Overall, the treatment proved highly effective, with 21 patients (80.8%) achieving complete healing and resuming oral intake after a median of 32 days (range 14–70). Treatment was unsuccessful in only five patients, including two with antral leaks and three with proximal leaks, and importantly, no procedure-related complications were observed [76]. A meta-analysis by S. Shoar et al. about efficacy and safety of the OTSC System in the management of leak and fistula after SG reported an overall successful closure rate of 86.3% (63/73 patients) for leaks and fistulas following SG using the OTSC system [77]. Although these results appear promising, they should be interpreted with caution due to several limitations. The limited number of cases available necessitated pooling data from heterogeneous studies and case reports, resulting in potential variability in patient selection criteria and treatment protocols. Furthermore, important variables remain unclear, such as the optimal timing for OTSC application and specific indications or contraindications. The generally short follow-up precludes assessment of long-term durability, and the difficulty in distinguishing the efficacy of the OTSC system alone from its use in combination with other endoscopic procedures further limits the interpretation of the findings [77].

##### Endoscopic Suturing

Endoscopic suturing represents an alternative technique for the management of post-anastomotic leaks. Its primary advantage lies in the potential for precise tissue approximation; however, the procedure demands additional training and expertise, as performing suturing in a confined endoluminal space or at angled sites can be technically challenging [78]. The OverStitch system (Apollo Endosurgery, Austin, TX, USA) has emerged as a leading endoscopic suturing platform. This single-use, disposable device allows for the placement of either permanent or absorbable full-thickness sutures [79]. The role of endoscopic suturing as a treatment for leaks following bariatric surgery has been addressed in only a few studies, which are generally limited by small and heterogeneous patient populations. R. Z. Sharaiha et al. analyzed a series of 20 patients, in whom the majority of anastomotic leaks occurred after SG (18/0, 90%), while a smaller proportion followed RYGB (2/20, 10%) [80]. Most patients (70%) had previously undergone treatments such as stenting (*n* = 10), fibrin glue (*n* = 4), or OTSC (*n* = 1). Additionally, 17 patients received concomitant therapies including stents (41.2%), APC (35%), fibrin glue (11.7%), and OTSC (11.7%). Endoscopic suturing achieved immediate technical success in 90% of cases (18/20). However, long-term clinical success was observed in only 27% of patients (95% CI, 8.6–49.1%). Success rates did not significantly differ between primary and rescue closure (*p* = 0.13), but long-term outcomes were more favorable when leaks were treated within 30 days of diagnosis compared with later interventions (44% vs. 0%, *p* = 0.041). These findings suggest that early intervention with endoscopic suturing may improve long-term outcomes, although overall success remains limited in a heavily pretreated population [80]. G. Fernandez-Esparrach et al. reported on the efficacy and safety of endoscopic suturing using the EndoCinch system (CR Bard, Murray Hill, NJ) for the treatment of gastrogastric fistulas in 71/95 patients who had undergone RYGB; the other 24/95 patients were treated with hemoclips [81]. The EndoCinch device was the most frequently used technique for endoscopic closure of gastro-gastric fistulas, accounting for 75% of treated patients. It was preferentially employed in cases with larger and, presumably, more complex fistulas, as demonstrated by the significantly greater mean diameter of lesions in this group compared with those managed with hemoclips (14.5 ± 8.7 mm vs. 7.7 ± 6.0 mm, *p* = 0.01). On average, 2.2 sutures were required per procedure (range 1–6), reflecting the need for a tailored approach based on fistula anatomy. Despite the greater technical demands and the larger size of fistulas treated, long-term closure rates did not significantly differ between EndoCinch and hemoclip groups (17% vs. 24%, *p* = 0.703). These findings suggest that endoscopic suturing with the EndoCinch, although technically demanding and often requiring multiple applications, remains a valid therapeutic option even in patients with larger and more challenging fistulas. Nevertheless, it is important to emphasize that in some cases more than one device was required to achieve effective closure [81].

##### Comparison of Endoscopic Closure Techniques

OTSC and endoscopic suturing have distinct advantages and limitations, which restrict their use to selected cases. The OTSC system offers high compressive strength, making it particularly suitable for closing small defects (<20 mm), and allows rapid deployment, which is advantageous in acute early leaks with viable, non-fibrotic margins [82]. However, its effectiveness is limited in larger defects, chronic leaks with epithelialized fistulas or persistent cavities, or when necrotic or fibrotic tissue is present. Placement can also be challenging in anatomically complex areas, and there is a risk of clip migration or dislodgement [82]. Consequently, OTSC is primarily indicated for small, localized leaks, often used in combination with endoscopic or percutaneous drainage when associated collections are present [17]. Endoscopic suturing (OverStitch and similar systems) offers greater technical flexibility, allowing closure of larger defects than OTSC and a more “surgical” approach through the application of multiple sutures and reinforcement of anastomoses or sleeves. This technique is particularly useful for subacute or late leaks, in cases where OTSC is not feasible or has failed, and in complex scenarios such as larger dehiscences or chronic fistulas. Endoscopic suturing can also be combined with other approaches, including stents, internal drains, or endoluminal vacuum therapy [17]. Disadvantages include greater technical complexity, longer procedural times, the need for dedicated equipment, higher costs compared to OTSC, and reduced effectiveness in chronic leaks with incompletely drained cavities [79].

#### 5.1.2. Self-Expanding Metal Stents (SEMSs)

The ESGE guidelines recommend that temporary endoscopic stent placement may be considered for the treatment of leaks, fistulas, and gastrointestinal perforations, without, however, indicating a specific type of stent and emphasizing that the duration of treatment should be individualized according to the clinical context [83]. In bariatric surgery, postoperative leaks represent one of the most feared complications, and endoscopic stenting has emerged as one of the main therapeutic approaches, often combined with percutaneous drainage of collections [84]. Stent placement enables so-called “diversion therapy,” which diverts luminal contents, reduces contamination of the dehiscence site, and facilitates earlier resumption of oral intake; in cases where longer prostheses are used, it is also possible to simultaneously treat distal gastric strictures, which may occasionally coexist [17]. Both partially covered self-expandable metal stents (PCSEMSs) and fully covered self-expandable metal stents (FCSEMSs) have been employed for this purpose, although each type presents distinct advantages and drawbacks [85]. FCSEMSs offer the advantage of easier removal but are associated with a higher risk of migration and incomplete sealing, as oral contents may leak alongside the device, preventing the establishment of a truly “watertight” seal [17]. In contrast, PCSEMSs provide greater stability and more effective diversion, reducing the risk of displacement, although their removal can be technically challenging [12]. Given that stent migration remains one of the most frequent complications of endoluminal stenting, various fixation and anchoring techniques have been developed to address this issue. These include external snare fixation, endoscopic suturing [86], and the application of endoscopic clips, either through-the-scope (TTS) or over-the-scope (OTSC), all of which have been shown to significantly reduce migration rates [87,88]. More recently, extra-long, fully covered SEMSs specifically designed for post-sleeve gastrectomy leaks—such as the MEGA esophageal stent (Taewoong Medical, Gyeonggi-do, South Korea) and the Hanarostent (MITECH, Seoul, South Korea), available in lengths up to 23 cm and 24 cm, respectively—have further lowered the incidence of stent-specific complications, including migration and difficulties during removal, providing a promising option for managing challenging post-bariatric leaks [87,89]. The role of self-expanding metallic stents (SEMSs) in the management of post-bariatric surgery leaks has been extensively analyzed in recent years. A. M. Hernández et al. published a meta-analysis that included 22 studies comprising a total of 488 patients who underwent stent placement for leaks following bariatric surgery [90]. The surgical procedure most frequently associated with the development of leaks was SG, followed by RYGB and duodenal switch, although the meta-analysis did not report absolute numbers for each technique. Only four studies reported data on fistula size, which was <10 mm in a minority of cases [90]. Prior to stent placement, many patients had already undergone conservative treatments, including fasting, enteral or parenteral nutritional support, antibiotics, and surgical or percutaneous drainage of collections. In some cohorts, more than 50% of patients had a drainage in place before the endoscopic procedure. All studies reported the use of covered stents, predominantly FCSEMS, with lengths ranging from 18 to 23 cm. The primary outcome, complete leak closure, was achieved in 85.9% of cases (95% CI 82.5–89.2%). The most frequent complications were stent migration, occurring in 18.6% of patients (95% CI 14.3–23.0%), and minor symptoms such as chest pain, nausea, vomiting, and reflux, which were generally self-limiting [90]. Severe complications, such as bleeding or perforation, were rare. The need for surgical reintervention was 13.5% (95% CI 9.9–17.1%), while overall mortality was 2.05% (10 deaths among 488 patients), none of which were directly related to stenting [90]. In summary, this meta-analysis demonstrates that the use of SEMS represents an effective and relatively safe strategy for the management of post-bariatric leaks, with high success rates and low mortality. However, the frequent need for combined treatments (drainage, nutritional support) and the non-negligible incidence of complications, particularly stent migration, highlight the importance of a multidisciplinary approach and the need to develop devices specifically designed for this clinical indication.

#### 5.1.3. Endoscopic Internal Drainage

**Figure 6:**
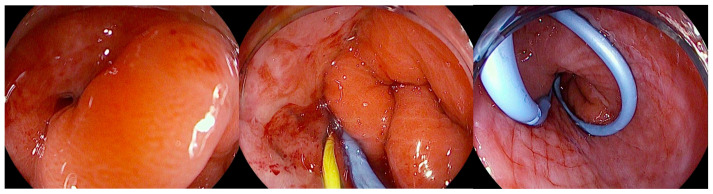
Endoscopic view of a gastric fistula after sleeve gastrectomy communicating with a perigastric cavity, showing the transfistulous endoscopic placement of a 0.035 Fr radiopaque guidewire followed by deployment of a double-pigtail plastic stent. The copyright of this figure belongs to the authors.

In patients who develop leaks or fistulas following bariatric surgery, it is increasingly recognized that techniques aimed at directly closing the defect do not always represent the optimal strategy, particularly once the lesion has become chronic and epithelialized. In such cases, promoting internal drainage by maintaining the fistulous tract open may be more effective [91]. This approach is based on the principle that, when intragastric pressure is lower than the pressure within the perigastric collection, fluid preferentially drains into the stomach, leading to progressive emptying and eventual collapse of the cavity [75]. Clinical success is therefore better assessed by dietary tolerance and reduction in the size of the perigastric collection rather than by the mere narrowing of the fistulous orifice. For endoscopic internal drainage (EID) to be effective as a stand-alone therapeutic strategy, however, certain conditions must be absent, including disorganized collections, distal stenosis resulting in elevated intragastric pressure, or the presence of a gastropleural fistula [92]. The concomitant use of percutaneous drains must also be carefully managed: continuous drainage lowers intracavitary pressure and prevents effective internal drainage into the gastric lumen. For this reason, such drains should remain clamped and only be opened during scheduled lavage procedures. Several endoscopic approaches can facilitate internal drainage, including the placement of short, small-caliber double-pigtail stents (DPSs) and endoscopic vacuum therapy (EVT). Among these, DPS is the most commonly adopted technique, with routine exchanges performed every 2–4 weeks until the perigastric cavity has nearly resolved while EVT applies continuous negative pressure to promote cavity closure [93]. Ultimately, the choice of technique should be individualized, and in many cases require a multimodal and sequential approach. The goal is not necessarily immediate fistula closure but rather the restoration of a favorable pressure gradient that facilitates gradual resolution of the perigastric cavity. DPS are plastic devices (most commonly made of polyethylene or polyurethane) specifically designed to facilitate internal drainage within the gastrointestinal tract. Their tubular structure is characterized by bilaterally curled ends (“pigtails”), which act as anchors to minimize the risk of migration both into the gastrointestinal lumen and into the drained cavity. They are available in a wide range of calibers, typically between 5 and 10 Fr, and lengths from 5 to 18 cm, with some models incorporating multiple lateral side holes to enhance drainage capacity, as well as radiopaque markers to ensure accurate positioning under fluoroscopic guidance. Deployment is generally performed over a 0.035-inch guidewire using radiopaque pusher catheters, which facilitate advancement even through tortuous tracts [94] (Figure 6).

The combination of flexibility, resistance to kinking, and secure anchoring makes DPS particularly effective for the drainage of pancreatic pseudocysts, postoperative collections, and anastomotic leaks, where continuous diversion of fluid into the gastrointestinal lumen promotes local sepsis control and progressive healing of the defect. This technical profile has been consistently reported in the literature, with high technical and clinical success rates across different indications. EID with DPS has emerged as one of the most effective and safest strategies for the management of post-bariatric surgery complications, both after RYGB and SG, with growing evidence supporting its role as a first-line therapy. The first systematic experience was reported by Donatelli et al. in a cohort of 33 patients with leaks after RYGB, of which 91% were located at the cardia along the proximal staple line and three at the gastrojejunal anastomosis [95]. The technique consisted of transfistulous placement of one or more DPS (Visio G-flex or Advanix Boston Scientific) ranging from 7 to 10 Fr in diameter and 3 to 10 cm in length, selected according to cavity size and tract characteristics, combined with a nasojejunal feeding tube to ensure early enteral nutrition. 60% of patients had undergone prior surgical drainage for sepsis control, while 40% were treated directly with endoscopy. Follow-up included an endoscopic reassessment at one month and subsequently monthly until healing. Outcomes were highly favorable: clinical success was achieved in 97% of cases, with a mean treatment duration of 61 days (range 28–99). The most frequent finding was the occurrence of a subclinical gastro-gastric fistula at the first follow-up, which was successfully managed with re-stenting. Only one patient (3%) experienced treatment failure due to ischemia and staple-line stenosis, and no patient required late surgical revision, strongly supporting the rationale of “drain and wait” over traditional “cover and close” approaches [95]. These findings were later confirmed and extended in SL. The systematic review by Giuliani et al. (2019) included 11 studies and 385 patients treated with DPS for leaks or fistulas after SG [96]. Overall, the closure rate was 83.4%, which rose to 84.7% when EID was used as a first-line therapy. Importantly, the safety profile was superior to other endoscopic techniques such as FC-SEMS, OTSC, or tissue sealants, which carried higher risks of migration, ulceration, and other AEs. This review highlighted the progressive evolution of EID from a salvage technique to a primary therapeutic option, although it also underscored the heterogeneity of stent sizes, calibers, and numbers across published series, pointing to the need for greater standardization [96]. A decisive contribution to defining the therapeutic standard came from the large monocentric series of Donatelli et al., which evaluated 617 consecutive patients with complications after SG treated at a referral center [97]. The cohort included 300 leaks (48.6%), 285 chronic fistulas (46.2%), and 32 isolated collections (5.2%). The therapeutic strategy consisted of transfistulous DPS placement for leaks and fistulas, while isolated collections were drained by EUS-guided DPS or, in selected cases, by lumen-apposing metal stent (LAMS). Follow-up was rigorously standardized, with scheduled reassessments at one and three months, and endotherapy discontinued if healing had not occurred within 120 days [97]. Results confirmed the high efficacy of the method: overall clinical success was 84.7%, with subgroup outcomes of 89.5% for leaks, 78.5% for fistulas, and 90% for collections. Recurrence was rare (1.8%), and AEs occurred in only 4.5% of cases, mostly manageable bleeding episodes, with occasional DPS migration. Predictors of failure included the presence of chronic fistula, use of combined endoscopic approaches, prior emergency surgery, and previous endoscopic treatments, thereby reinforcing the indication for early and standardized EID as the best means of maximizing outcomes [97]. Finally, the comparative study by Lorenzo et al. provided the clearest demonstration of the superiority of EID over closure-based strategies. In this analysis of 100 SG-related fistulas, patients were treated either with DPS (often combined with nasocavitary drains) or with closure/bridging techniques using FC-SEMSs and/or clips [98]. Outcomes were striking: primary success was 86% with EID versus only 63% with closure, with an overall success rate of 86%. In patients with collections larger than 5 cm, EID was clearly superior, whereas SEMS placement was associated with an exceedingly high complication rate, with stent migration or peri-stent ulceration in up to 50% of cases [98]. Additional negative prognostic factors included prior surgical reintervention before endoscopic therapy and the presence of purulent drainage at endoscopy, both of which were associated with prolonged healing times exceeding six months. This study marked the turning point for the Marseille group, which, from 2013 onward, transitioned from a SEMS-based closure strategy to the “drain and wait” paradigm, now widely adopted in high-volume bariatric centers [98].

#### 5.1.4. Endoscopic Vacuum Therapy

EVT is now a key treatment for post-esophagectomy anastomotic leaks [93,99]. First used about 15 years ago for colorectal leaks, it has since been adapted for the esophagus and upper GI tract. The technique uses a polyurethane sponge attached to a vacuum system, typically set at −125 mmHg, though no formal guidelines exist. Negative pressure promotes healing by removing infected secretions, improving perfusion, and stimulating granulation tissue [93]. The sponge is usually replaced every 3–5 days to maintain efficacy and prevent complications such as infection or tissue adherence. EVT can be applied intracavitarily (within the leak cavity, preferred for larger leaks with collections) or intraluminally (within the lumen, across the defect, suitable for smaller leaks without collections) [93]. To date, no studies directly compare the outcomes of these two approaches. The available literature on EVT for the management of post-bariatric surgery leaks remains extremely limited. Most of the evidence derives from small, retrospective case series with very few patients, often reported by single high-volume centers. Prospective studies and randomized controlled trials are lacking, which makes it difficult to draw definitive conclusions regarding the true effectiveness of the technique or its comparative value against alternative strategies such as SEMS, OTSC, or EID. Within this context, the systematic review and meta-analysis published by J. M. V. Intriago et al. in 2022 represents the first attempt to synthesize the available data [100]. The analysis included five studies (four retrospective case series and one cohort), enrolling a total of 55 patients with post-bariatric leaks or fistulas [100]. EVT was used as a first-line treatment in only about 32.7% of cases, while in the majority it served as a rescue option following failure of surgical or conventional endoscopic approaches. Pooled results showed a clinical success rate, defined as fistula/leak closure) of 87.2%, with a favorable safety profile: moderate AEs occurred in 6% of patients, device dislodgement in 12.5%, and no fatalities were directly attributable to EVT [100]. Nevertheless, EVT is associated with a considerable procedural burden. On average, patients required 6.47 device exchanges (95% CI 4.00–8.94; I^2^ = 85.30%; *p* < 0.001) at intervals of 4.39 days (95% CI 3.60–5.17; I^2^ = 93.31%; *p* < 0.001). The mean duration of treatment was 25.6 days (95% CI 15.16–36.18; I^2^ = 93.31%; *p* < 0.001), with a prolonged hospital stay averaging 44.4 days (95% CI 30.01–58.84; I^2^ = 80.82%; *p* < 0.001). In addition, two studies reported the use of adjunctive therapy during EVT, primarily laparoscopic interventions, with a pooled rate of 35.3% of patients requiring additional procedures (95% CI 19.3–75.2; I^2^ = 14.6%; *p* = 0.284). Despite these challenges, current evidence supports EVT as a safe and effective therapeutic option in this setting [100]. Subsequent studies have further reinforced the promising outcomes of EVT in the management of post-bariatric surgery leaks. Kollmann et al. published a retrospective single-center cohort [101]. Out of 1046 bariatric procedures, 17 patients developed gastric leaks (1.0%), of whom nine received EVT as first-line therapy and eight after failure of alternative interventions such as revisional surgery or stent placement [101]. Remarkably, EVT achieved a 100% closure rate with no treatment-related mortality. Patients treated with primary EVT had significantly shorter treatment duration (median 17 days) and hospital stay (median 36 days) compared with those who underwent secondary EVT (61 and 68 days, respectively), underlining the importance of early initiation of EVT [101]. Similarly, Gensthaler et al. reported a retrospective single-center experience, including 21 patients treated with EVT for anastomotic or staple-line leaks [102]. EVT was performed either as a standalone intervention (14.3%) or in combination with revisional surgery and, in many cases, adjunctive SEMS placement (76.2%). The therapy required a median of six sponge changes with an average dwell time of 25.7 days, but it resulted in a clinical success rate of 95.2%, with no severe EVT-related complications [102]. Both studies, despite their small sample sizes and retrospective design, consistently demonstrate that EVT offers high efficacy and safety in managing leaks after bariatric surgery, further supporting the findings of the earlier meta-analysis.

A recent systematic review and meta-analysis by Laopeamthong et al. has provided a comparative evaluation of the two most widely adopted endoscopic strategies for post-bariatric leaks, namely EVT and EID [103]. The authors included 13 studies with a total of 279 patients (3 EVT studies and 10 EID studies) and performed a proportional meta-analysis on closure rates. Both modalities achieved high clinical success, with pooled closure rates of 85.2% (95% CI 75.1–95.4%) for EVT and 91.6% (95% CI 88.1–95.2%) for EID, suggesting that either method can be considered effective as a first-line endoscopic option. Interestingly, the mean duration of treatment was considerably shorter with EVT (28 days, 95% CI 2.4–53.6) compared to EID (78.4 days, 95% CI 50.1–106.7), although the confidence intervals were wide, reflecting heterogeneity in treatment protocols across studies. Data on AEs, recurrence, or the need for additional procedures were limited and inconsistently reported, underscoring the lack of standardization in the available literature [103]. Despite these limitations, the findings of this meta-analysis reinforce the concept that both EVT and EID are valid first-line strategies, with EID showing a slightly higher pooled success rate, while EVT may offer faster closure in selected patients. The study also highlights the urgent need for larger, prospective comparative trials to better delineate optimal patient selection, technical protocols, and long-term outcomes of these two evolving approaches. Table 2 shows the results of the main studies on the efficacy and safety of the principal studies cited in the meta-analysis (Table 2).

The VAC-Stent is a novel device that integrates features of SEMS and EVT. It consists of a fully covered nitinol SEMS with a silicone membrane, around which a polyurethane sponge is wrapped, connected via a catheter to a vacuum pump. Its mechanism of action suggests a potential role particularly in cases without large extraluminal cavities, but its exact place in the therapeutic algorithm has yet to be defined [110]. Although early reports describe successful use of the VAC-Stent in bariatric surgery, esophageal perforations, and even prophylactically during esophagectomy in high-risk patients, the available evidence remains scarce. To date, only a few case reports have been published regarding its application in the management of post-bariatric leaks, yet these have shown encouraging results, suggesting a potential role for this device in future clinical practice [111,112,113].

#### 5.1.5. EUS-Guided Drainage of Collections

Endoscopic ultrasound (EUS) has emerged as a key innovation in the management of post-bariatric surgery complications, particularly leaks, fistulas, and perivisceral collections. Collections, defined as accumulations of fluid or infected material adjacent to the operated gastrointestinal tract, may be either isolated or in communication with the lumen, and require a tailored therapeutic strategy. EUS provides not only more accurate diagnostic capabilities but also precise localization of the collection in relation to the gastric or duodenal wall, while simultaneously assessing vascular structures. This aids in transmural drainage and also helps reduce AEs like bleeding and perforation [114]. This approach allows determination of whether the collection is well demarcated and amenable to a transmural intervention, thereby enabling safe and effective endoscopic drainage. Such drainage can be performed using plastic stents or LAMS, which create direct and stable communication between the cavity and the gastrointestinal tract. While EUS-guided drainage has become a well-established and widely adopted therapeutic strategy in the management of pancreatic fluid collections, its role in the treatment of collections arising after bariatric surgery is still being defined and progressively codified [115]. In this specific clinical setting, post-surgical collections often present unique anatomical and technical challenges, related both to the altered gastrointestinal anatomy and to the frequent presence of concomitant complications such as leaks or fistulas. Consequently, although the principles and techniques of EUS-guided drainage are shared with those applied in pancreatology [116], their application in bariatric surgery requires careful patient selection, thorough pre-procedural assessment, and expertise in advanced endoscopy. Growing evidence suggests that EUS-guided drainage provides a minimally invasive, effective, and safer alternative to surgical or percutaneous approaches, but further studies and standardized protocols are still needed to consolidate its role and optimize outcomes in this specific post-surgical population.

The first evidence regarding the use of EUS-guided drainage comes from mixed cohorts, in which bariatric patients represented a subgroup. Mudireddy et al. reported a multicenter experience including 47 patients with postoperative collections (of various origins, including bariatric) treated with EUS-guided LAMS. Technical success was achieved in 93.6% of cases and clinical success in 89.3%, while AEs occurred in 6.4%, all of which were manageable either endoscopically or conservatively [114]. Similarly, Terrin et al. published a series of 66 postoperative collections treated with EUS-guided LAMS: technical success was 97% and clinical success 95.2%, with only one severe AEs (1.5%). The authors emphasized that outcomes were independent of surgical type, timing, or collection size, thereby supporting the robustness of the technique, including in the bariatric subgroup [117]. In a series by Donatelli et al. including 32 postoperative collections (5 bariatric), EUS-guided drainage was the most frequently adopted approach, with either multiple DPSs or LAMSs depending on the cavity. Clinical success reached 93.4%, with an AEs rate of 12.5%, predominantly bleeding managed with conservative or radiological therapy [118]. In studies specifically addressing bariatric surgery, data are more detailed. In 2021, Donatelli et al. reported the experience of a referral center that managed 1020 patients with post-bariatric surgery complications, of whom 33.2% presented with fistulas, 31.8% with leaks, and 3.6% with postoperative collections [97]. In this latter subgroup, EUS was mainly employed for drainage, achieving a clinical success rate of 88.2%. The team applied an internal protocol, not standardized at the international level, which consisted of EUS-guided puncture with a 19G needle, tract dilation, and stent placement: either multiple DPS, left in place for approximately three months, or LAMS, which were systematically removed or replaced with DPS after one month [119]. Indications were limited to collections adjacent to the gastrointestinal lumen and accessible endoscopically, with management complemented by enteral nutrition via nasojejunal tube and EID. Across the entire cohort, endoscopic procedures were associated with a 1.9% rate of AEs, including perforations, bleeding, stent migration [119]. This approach, while achieving favorable clinical outcomes, reflects a local protocol developed by the center’s experience rather than a codified practice endorsed by international guidelines, underlining the need for further studies to define standardized strategies [119]. In the largest series, Donatelli et al. analyzed 617 post-SG complications: 300 leaks, 285 fistulas, and 32 collections. EUS was used exclusively for intra-abdominal collections (5.2% of the cohort), where either DPS (*n* = 28) or LAMS (*n* = 2) were placed. Clinical success for collections was 90%, with an overall AEs rate of 4.5% (mainly bleeding, splenic pseudoaneurysms, pneumoperitoneum) and recurrence in 1.8% of cases [97]. Smaller experiences have described complex scenarios. Machlab et al. reported a case of septic subphrenic collection after SG, not amenable to percutaneous drainage: an EUS-guided transgastric drainage with a 15 × 10 mm LAMS was performed, resulting in rapid clinical improvement. The stent was removed after 2 weeks, and follow-up endoscopy at 6 weeks confirmed complete closure of the defect [120]. Lajin et al. described a chronic refractory microfistula after SG treated with LAMS combined with DPS, followed by endoscopic septotomy; complete healing was achieved after cavity epithelialization [121]. Similarly, Lippai et al. reported the role of EUS-guided drainage in the setting of a gastrocutaneous fistula where the internal orifice was no longer identifiable endoscopically; EUS allowed access to the cavity and placement of a DPS, leading to clinical resolution [122]. In summary, current evidence indicates that EUS-guided drainage represents a reliable therapeutic option in complex scenarios, with advantages in cases where direct access to the defect is not feasible or in patients with organized collections not amenable to percutaneous drainage.

### 5.2. Post-Surgical Strictures

Endoscopic dilation (ED) and the placement of SEMS are the most frequently reported endoscopic techniques in the literature for the management of post-surgical strictures [12]. ED can be performed using either mechanical bougie dilators or pneumatic balloon dilators (BD). Mechanical dilators are further categorized according to whether guidewire placement and/or fluoroscopic guidance is required. Non-guidewire-dependent bougies, such as Maloney dilators, are weighted with mercury or tungsten and feature a tapered tip, and they are available in a wide range of sizes to accommodate different clinical scenarios. The most used guidewire-assisted mechanical bougie is the polyvinyl Savary-Gilliard dilator (Cook Medical, Billerica, MA, USA). Through-the-scope BD are also available in both guidewire-assisted and non-guidewire-assisted designs [123]. Radially expanding BD are available in multiple designs, sizes, and lengths for treating strictures throughout the gastrointestinal tract. They can be advanced through the endoscope with or without guidewire assistance, allowing direct visualization of the procedure. These balloons provide uniform and controlled expansion, either to a single fixed diameter or sequentially to increasing diameters. Inflation is achieved by injecting liquid under pressure, with manometric monitoring of balloon force; radiopaque contrast may be added to enhance fluoroscopic visualization [123]. Achalasia BDs represent a specialized subtype: large-diameter polyethylene balloons (30–40 mm), exclusively wire-guided and disposable. They cannot be passed through the endoscope and must be positioned fluoroscopically, with placement confirmed by radiopaque markers and inflation monitored manometrically [124]. In the management of post-SG strictures, pneumatic BD represent the most employed devices.

#### 5.2.1. Endoscopic Management of Strictures After SG

Strictures after SG were broadly classified as functional, when related to a sleeve twist without structural narrowing; organic, when due to a mechanical obstruction with evident luminal reduction; or combined, when both mechanisms occurred together. Twisting of gastric tube is recognized as one of the main functional causes of persistent reflux and food intolerance following SG. It consists of an axial rotation of the gastric tube, which during endoscopy appears as a clockwise deviation of the staple line, typically at the level of the incisura angularis [125]. In contrast, a normally configured sleeve shows a straight and symmetrical staple line without deviations [126]. To date, no universally accepted classification of this condition has been established. The only attempt proposed in 2022 by L. T. Siqueira et al., introduced an endoscopic grading system with three degrees of torsion. In Grade I, a mild rotation is observed without significant luminal narrowing; in Grade II, the rotation is more pronounced, producing a fixed area of narrowing that requires additional endoscopic maneuvers to be traversed; and in Grade III, the torsion is severe, resulting in true stenosis with marked difficulty or even impossibility of endoscopic passage [127]. Since SG stenosis is relatively uncommon, standardized management guidelines are lacking. Reported approaches range from conservative strategies, such as observation and bowel rest, to surgical options including revisional SG, seromyotomy, or conversion to RYGB [128]. Endoscopic techniques, given their favorable safety profile, have been proposed as the first-line treatment and should be considered prior to major surgical interventions. There is currently no standardized therapeutic algorithm for post-SG stenosis. In 2023, A. D’Alessandro et al. published the largest retrospective series to date, including 202 patients, and proposed a treatment strategy tailored to the type and length of stenosis (inflammatory 4.5%, fibrotic 11%, and functional 84.5%) [129]. The algorithm suggested that early, long inflammatory strictures be initially managed with a 10F nasojejunal tube for four weeks, followed by gradual refeeding; patients achieving symptom resolution were considered cured. Persistent cases were subsequently treated according to their classification (fibrotic or functional). For short, non-inflammatory strictures, either pneumatic BD or placement of a LAMS 4 weeks) was recommended, with LAMS preferred within the first month after surgery due to the higher perforation risk associated with dilatation [129]. Patients showing only partial improvement or recurrence underwent progressive pneumatic dilatation with balloons up to 40 mm. In cases of long fibrotic or functional strictures, treatment options included pneumatic dilatation or temporary placement of an FC-SEMS. Recurrences were managed with repeat dilatation or replacement of the stent. Therapeutic strategies were heterogeneous and tailored to patient characteristics: pneumatic dilation, either single or progressive, represented the most frequently performed intervention (116 patients in total), while stent placement (LAMS in 35 cases, FC-SEMS in 8) or combined approaches (dilation plus stent in 36 cases) expanded the range of available treatments [129]. The endoscopic approach achieved an overall success rate of 69% at two-year follow-up. Efficacy varied according to the type and location of stenosis: the highest outcomes were reported for inflammatory strictures (up to 100% in medium strictures), followed by functional strictures (73.5–85.7%) and pure fibrotic strictures (71.4–81.8%). Regarding safety, only four major AEs were recorded (three gastric perforations and one bleeding episode, all related to pneumatic dilation in patients with gastric twist), which were successfully managed either conservatively or surgically. No major complications were reported with LAMS or FC-SEMS [129]. Minor AEs included stent migration (3%), intolerance to FC-SEMS, and retrosternal pain after dilation, all managed conservatively. Overall, these findings confirm that endoscopy should be considered the first-line therapeutic option, offering both effectiveness and safety while significantly reducing the need for revisional surgery (135). A recent meta-analysis by Eguchi et al. (2025), which encompassed 28 retrospective studies with a total of 1068 patients, gastric strictures were most frequently located at the incisura (70%) and, to a lesser extent, at the gastroesophageal junction (17%) [130]. The authors categorized strictures into three main types: functional, characterized by a torsion of the gastric sleeve without true anatomical narrowing; organic, defined by a clear mechanical obstruction; and mixed forms, in which both mechanisms coexist. The response rate to ED was approximately 77.8% (95% CI 68–85.3; I^2^ = 71%), with an average of 1.82 dilations per patient (95% CI 1.6–2; I^2^ = 77%). Stent placement was required in 19.3% of cases (95% CI 12.9–27.7; I^2^ = 69%), while 16% of patients subsequently needed surgical intervention (95% CI 10.6–23.3; I^2^ = 69%). The mean post-procedural hospital stay was 3.9 days (95% CI 3.5–4.2; I^2^ = 64%), which is significantly shorter than that reported for surgical revision procedures. It is important to note that the I^2^ values indicate substantial heterogeneity across studies, meaning there was considerable variability in patient populations, procedural techniques, and study designs. The most used technique was pneumatic BD, reported in 21 studies. Balloon diameters ranged from 30 to 40 mm. Procedures typically followed a step-up approach, gradually increasing the balloon size from 30 to 35 and then 40 mm, with intervals of 2–4 weeks between sessions. Self-expanding stents, including LAMS and FC-SEMS, were primarily reserved for cases where balloon dilation alone was insufficient. These stents achieved success rates of up to 70% and were generally indicated for recent strictures (less than four weeks old) or longer strictures exceeding 3 cm. Based on the data gathered from their meta-analysis, the authors sought to translate the pooled outcomes into a practical, stepwise treatment algorithm for managing SG stenosis. By analyzing the effectiveness and limitations of various endoscopic approaches, they aimed to provide clinicians with a structured framework to guide decision-making. The proposed algorithm begins with BD as the first-line therapy, escalates to repeated dilations using a step-up strategy if needed, and reserves stent placement for cases unresponsive to serial dilations. Surgical intervention is considered only when endoscopic measures fail, emphasizing a graduated approach that balances efficacy with safety and minimally invasive management [130].

#### 5.2.2. Endoscopic Management of Strictures After RYGB

Gastrojejunal anastomotic stricture is one of the most frequent complications seen after RYGB. Endoscopic balloon dilation is the primary treatment for anastomotic strictures following RYGB performed for bariatric surgery. This technique is widely recommended because of its strong safety and efficacy profile, with gradual dilation using through-the-scope balloon dilators considered the preferred approach [131]. A meta-analysis by Baumann et al. including 21 observational studies with a total of 896 patients with anastomotic stenosis following RYGB [132]. Endoscopic management was the predominant approach, with TTS balloon dilation being the main technique; only 51 patients were treated with Savary-Gilliard bougies, while a single study included 26 patients who received adjunctive steroid therapy. ED was the primary intervention, with balloon diameters initially ranging from 9.37 to 17.71 mm and reaching a maximum of 25 mm. Patients generally underwent multiple sessions, with an average of 4.4 procedures per patient, and just over a third requiring more than one session [132]. Overall, endoscopic treatment proved highly effective, resulting in symptomatic improvement in 97% of patients (95% CI: 94–98). Complications were relatively uncommon, occurring in 4% of cases (95% CI: 3–6), and were predominantly perforations, most of which were managed conservatively. Only a small number of patients required surgical intervention (eight patients) or revision (five patients), while associated anastomotic ulcers were identified in 6% of cases (95% CI: 3–12). Surgery due to dilation failure was necessary in only 3% of patients. Predictors of treatment outcomes were closely related to the initial dilation parameters. A larger maximum diameter achieved during the first dilation was strongly associated with clinical success (OR 1.36; 95% CI: 1.11–1.70) and reduced the likelihood of requiring subsequent surgery (OR 0.74; 95% CI: 0.60–0.90) [132]. Similarly, the minimum diameter used during the first session was inversely correlated with the need for additional procedures (OR 0.86; 95% CI: 0.75–0.98) and with the risk of complications, suggesting that selecting an adequate initial diameter can significantly improve both safety and efficacy of endoscopic management [132]. Overall, these findings support that endoscopic dilation is a safe and highly effective first-line treatment for anastomotic stenosis after RYGB, providing substantial symptomatic relief with a low risk of serious complications and minimizing the need for surgical intervention. Endoscopic stents play a crucial role not only in the management of anastomotic fistulas but also in the treatment of refractory strictures [133]. Several therapeutic options are available for refractory strictures, including endoscopic incisional therapy (EIT) and stent placement. EIT entails incision and/or excision of the fibrotic ring causing the narrowing, most commonly performed with electrocautery [134]. Available evidence suggests that EIT is a feasible and potentially effective approach, particularly in treatment-naïve, short strictures, where it has been associated with lower recurrence rates compared with dilation alone. Conversely, its performance appears less consistent in longer or recurrent strictures, and repeated sessions are often necessary to achieve sustained patency [135]. These data should be interpreted cautiously, as published studies include heterogeneous populations and clinical contexts, and prospective investigations specifically focused on refractory post-bariatric strictures are lacking. Accordingly, while the literature is encouraging, the external validity of current findings remains uncertain and further indication-specific prospective studies are warranted [12]. Stent placement is a widely adopted approach for addressing refractory benign gastrointestinal strictures, particularly in cases that remain unresponsive after a minimum of five ED sessions over a two-week period [83]. However, data specifically evaluating the use of FC-SEMS for the management of strictures following RYGB are still limited. LAMSs are bi-flanged, dumbbell-shaped FC-SEMS originally designed for EUS-guided transluminal drainage [116]. Their use has since expanded to include benign gastrointestinal strictures [116]. LAMSs provide several advantages, including variable diameters and lengths, a saddle-shaped design with wide flanges that improve anchorage and reduce migration, and a simple deployment process that facilitates successful stent placement. Additionally, the gradual radial force exerted during expansion contributes to stricture resolution [12]. Although clinical evidence remains limited, early data suggest that LAMS may be a safe and effective option for managing refractory luminal strictures. The use of LAMS as a therapeutic approach for anastomotic strictures following RYGB has been evaluated in several recent studies, both as a first-line treatment and as a second-line option after multiple sessions of endoscopic dilation. In all cases, LAMS demonstrated high efficacy and safety, proving effective for both newly diagnosed and refractory strictures [136,137,138]. *A.* Spota et al. published a retrospective study on 1020 patients treated for complications of bariatric surgery, applying a standardized endoscopic algorithm for the management of various conditions [119]. Within this cohort, 95 patients (9.3%) presented with an anastomotic stricture after RYGB. Stricture management followed a defined pathway: sequential ED up to 15 mm every 30 days in less severe cases, while temporary placement of LAMS (16 × 30 mm, maintained for 4 weeks) was used for fibrotic or cicatricial strictures. The algorithm achieved a clinical success rate of 81.5%, with restoration of regular oral intake, whereas 16.8% required surgical reintervention and 3.2% were still under treatment at the end of follow-up [119]. This work demonstrates that an early and systematic endoscopic approach, based on a shared algorithm, ensures high effectiveness rates and significantly reduces the need for surgical revision [119].

## 6. Discussion and Future Directions

**Figure 7:**
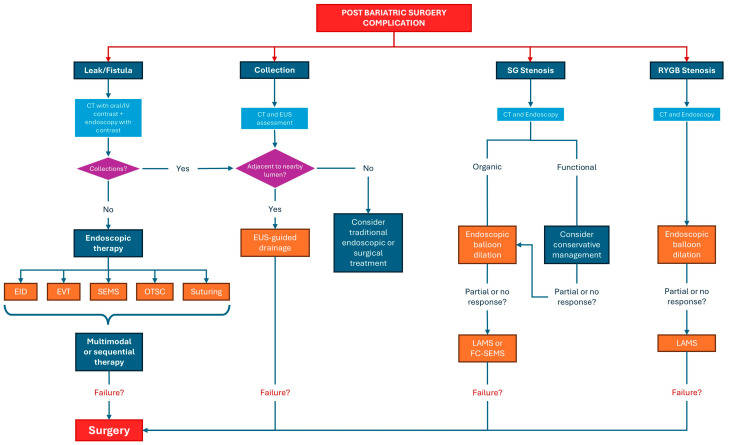
Algorithm that briefly summarizes the most consistent evidence that should guide the diagnostic, clinical and therapeutic management of complications after bariatric surgery.

Post-bariatric complications, although relatively uncommon, carry a significant clinical and prognostic impact. The incidence of leaks ranges from 1% to 6% depending on the surgical technique, with higher rates after RYGB compared to SG. Overall mortality remains low (<1%), yet among patients who develop a leak, mortality may reach 15–17%, highlighting the importance of early diagnosis and prompt intervention. Anastomotic strictures are reported in 3–27% of RYGB cases, whereas post-SG strictures are less frequent (0.5–3.5%), but nonetheless represent a major source of symptoms and hospital readmissions. Several risk factors have been identified as determinants of postoperative complications. Preoperative factors include high BMI, advanced age, comorbidities such as diabetes, hypertension, chronic kidney disease, obstructive sleep apnea, hypoalbuminemia, and chronic corticosteroid therapy. Intraoperative risk factors include a short distance from the pylorus, excessive narrowing or torsion of the gastric tube, and the use of surgical drains, all of which have been associated with increased rates of leaks and strictures. Smoking significantly increases the risk of dehiscence, particularly after SG. These findings emphasize that prevention begins with careful patient selection and meticulous surgical technique. Against this epidemiological background, endoscopic management has become the cornerstone of treatment, offering minimally invasive and personalized strategies based on the type and timing of the complication. Primary closure techniques, such as OTSC and endoscopic suturing, have demonstrated high success rates in acute and small defects, with closure rates between 80–86% in selected cases. Nevertheless, their efficacy is limited in chronic fistulas, in the presence of ischemic tissue, or when cavities are not adequately drained. SEMS historically represented the standard of care, achieving closure rates around 86%, but are associated with frequent complications, including migration (≈18%) and peri-stent ulceration. As a result, clinical practice has shifted from the “cover and close” paradigm to the “drain and wait” approach. In this context, EID with DPS has emerged as a first-line therapy, with clinical success exceeding 85% and a favorable safety profile. EVT, adapted from the esophageal setting, has shown comparable efficacy, with success rates approaching 90–95% and faster healing times (25–30 days). However, EVT requires multiple sponge exchanges and prolonged hospital stays, limiting its widespread adoption. Comparative studies suggest that EVT is particularly advantageous in acute, large leaks with septic cavities, whereas EID is better suited for chronic fistulas and organized collections. EUS-guided drainage has further expanded the therapeutic arsenal, providing a minimally invasive option for post-surgical collections that are not amenable to percutaneous access. With clinical success rates of 90–95% and manageable adverse events, EUS-guided drainage with LAMS or DPS has become a reliable alternative, though standardized protocols regarding timing, device selection, and duration remain lacking. Similarly, post-surgical strictures have benefited from the endoscopic approach. Pneumatic balloon dilation remains the first-line treatment, achieving >95% success after RYGB with a perforation risk below 5%. In SG, strictures—often localized at the incisura angularis—respond in 70–80% of cases, though repeated sessions are frequently required. Refractory strictures may benefit from temporary LAMS or SEMS placement, with clinical resolution rates of 70–80%, significantly reducing the need for surgical revision. Despite these promising results, the current literature is limited by retrospective design, small sample sizes, heterogeneous populations, and short follow-up. Moreover, the absence of randomized controlled trials and international consensus guidelines hampers the standardization of diagnostic and therapeutic algorithms. Figure 7 presents an algorithm that provides an overview of the most consistent evidence available in the literature, integrating diagnostic, clinical, and therapeutic considerations to support the management of complications after bariatric surgery (Figure 7).

**Figure 8:**
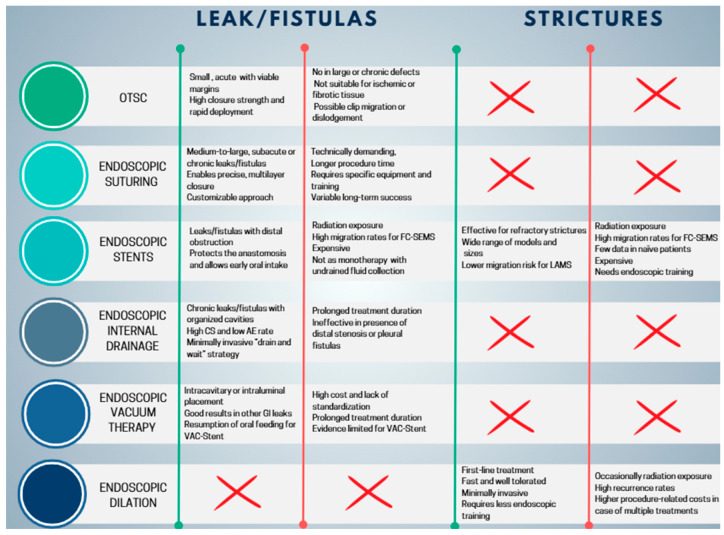
Pros and cons of endoscopic treatments in the management of post bariatric surgery complications. OTSC, Over-The-Scope Clip; FC-SEMS, fully covered self-expandable metal stents; LAMS, lumen-apposing metal stent; CS, clinical success; AE, adverse event; GI, gastrointestinal.

Future perspectives include the development of hybrid devices such as the VAC-Stent, the optimization of sequential multimodal strategies, and the use of artificial intelligence to enhance early diagnosis and patient stratification. Overall, endoscopic management of post-bariatric complications should no longer be regarded as a rescue option but as a structured, multidisciplinary component of care, with the potential to substantially improve survival and quality of life. Figure 8 provides an overview of the main benefits and limitations of endoscopic interventions for the management of postoperative leaks, fistulas, and strictures after bariatric surgery (Figure 8).

## 7. Conclusions

Post-bariatric complications, while infrequent, represent potentially life-threatening conditions that demand prompt recognition and tailored management. Endoscopic techniques have transformed their treatment, offering effective, safe, and minimally invasive alternatives to surgical reintervention. Current evidence highlights the high success rates of internal drainage with DPS, pneumatic balloon dilation, and EVT, underscoring the central role of endoscopy in this clinical setting. Nevertheless, the literature remains limited by the lack of prospective, randomized data and by heterogeneous protocols, which hinder the establishment of standardized therapeutic algorithms. The next steps involve multicenter collaborations, long-term outcome studies, and the integration of novel technologies, including hybrid devices and advanced imaging. The incorporation of artificial intelligence into diagnostic and therapeutic pathways may further optimize patient selection and outcomes. In summary, endoscopic management has become the preferred approach for most post-bariatric complications. Its progressive standardization and technological refinement will be crucial to ensuring even safer, more effective, and personalized care for this growing patient population.

## Figures and Tables

**Table 1:** Suggested CT Protocol for Post-Bariatric Surgery Complication Imaging.

Parameter	Protocol Recommendation
**Clinical Indications**	Suspected anastomotic leak, abscess, fistula, internal hernia, stricture, hemorrhage, or bowel obstruction
**Timing**	As clinically indicated, typically within first 10 days post-op for leaks; anytime for late complications
**Oral Contrast**	Water-soluble (e.g., amidotrizoate), 50–100 mL diluted in water, administered 30 min before scanning
**IV Contrast**	Non-ionic iodinated agent, 100–120 mL at 3–4 mL/s; bolus tracking or fixed delay (~65–70 s) for portal phase
**Scan Phases**	Non-contrast scan and portal venous phase standard; add delayed phase (90–120 s) if collections or fistulas suspected
**Patient Positioning**	Supine; consider prone or left lateral decubitus for suspected internal hernia or non-diagnostic supine scan
**Scan Coverage**	Entire abdomen and pelvis: diaphragm to pubic symphysis, including pouch, anastomoses, bowel, and mesentery
**Slice Thickness and Recon**	1–2 mm axial slices; coronal and sagittal MPRs essential for surgical anatomy and complication mapping
**Key Diagnostic Targets**	Extraluminal contrast, perianastomotic fluid or gas, mesenteric swirl, obstructed bowel, JJ displacement

**Table 2:** Major studies with the largest cohorts included in the reference meta-analysis. * EID and EVT are employed as primary endoscopic treatments, frequently in combination with adjunctive procedures (e.g., enteral feeding, stent placement, or surgical drainage). AEs, adverse events; SG, sleeve gastrectomy; RYGB, Roux-en-Y gastric bypass; EVT, endoscopic vacuum therapy; EID, Endoscopic internal drainage; SEMS, Self-expanding metal stents.

Authors, Years	Study Design	Bariatric Surgery	Intervention *	Clinical Success	AEs Related to the Endoscopic Procedure	Additional Treatments After Clinical Failure
Mencio M. A. et al., 2018 [104]	Retrospective					
		SG 17	EVT 18	14/17 (82.3%)	-	Surgery 3/17 (17.7%)
		RYGB 1		1/1 (100%)	-	-
Archid R. et al., 2020 [105]	Retrospective					
		SG 8	EVT 8	7/8 (87.5%)	Bleeding 1/8 (5.5%)	SEMS then surgery 1/8 (12.5%)
Donatelli G., et al., 2015 [106]	Retrospective					
		SG 64	EID 64 (9 under treatment)	50/64 (78.2%)	Pneumo-Peritoneum 2/64 (3.1%)	Surgery 3/64 (4.7%)
					Septic shock 1/64 (1.6%)	Cyanoacrylate glue 2/64 (3.1%)
Nedelcu M. et al., 2015 [107]	Retrospective					
		SG 9	EID 9	9/9 (100%)	-	Surgery 1/9 (11.1%)
Rebibo L. et al., 2016 [108]	Retrospective					
		SG 47	EID 47	43/47 (91.6%)	Migration 2/47 (4.3%)	Surgery 2/47 (4.3%)
					Perforation 1/47 (2.1%)	SEMS 2/47 (4.35)
Donatelli G. et al., 2017 [95]	Retrospective,					
		RYGB 33	EID 33	32/33 (97%)	-	Surgery 1/33 (3%)
Sportes A. et al., 2019 [109]	Retrospective,					
		SG 49	EID 49	41/49 (83.7%)	Sepsis 8/49 (16.3%)	Surgery 8/49 (16.3%)
					Bleeding 1/49 (2%)	

## Data Availability

No new data was created or analyzed in this study. Data sharing is not applicable to this article.
